# There's an app for that, but nobody's using it: Insights on improving patient access and adherence to digital therapeutics in Germany

**DOI:** 10.1177/20552076221104672

**Published:** 2022-07-03

**Authors:** Florian Dahlhausen, Maximillian Zinner, Linn Bieske, Jan P Ehlers, Philip Boehme, Leonard Fehring

**Affiliations:** 1 Faculty of Health, 12263 Witten/Herdecke University, Witten, Germany; 2 Didactics and Educational Research in Healthcare, Medical Department, Faculty of Health, 12263 Witten/Herdecke University, Witten, Germany; 3 Helios University Hospital Wuppertal, Clinic for Gastroenterology, Hepatology, Endocrinology and Diabetology, Heusnerstr. 40, 42283 Wuppertal, Germany

**Keywords:** Mobile health, mHealth, digital health, apps, digital therapeutics, DTx, reimbursement, adoption, adherence, healthcare professionals

## Abstract

**Background:**

Mobile health applications and their subset digital therapeutics—defined as evidence-based software interventions to prevent, manage, or treat a medical condition—offer great potential to improve patient care. However, such solutions often struggle to reach widespread adoption.

**Objective:**

This study seeks to explore healthcare stakeholders’ roles and potential for fostering patient access and adherence to evidence-based digital therapeutics and thereby improve health outcomes from the perspective of digital therapeutics developers and distributors.

**Methods:**

Semi-structured qualitative and semiquantitative interviews were conducted with 19 experts from developers and distributors of digital therapeutics in Germany to discuss their perceived relevance of different healthcare stakeholders and strategies in promoting patient access and adherence to digital therapeutics.

**Results:**

Healthcare professionals were found to have the greatest potential to promote both access and patient adherence to digital therapeutics, followed by health insurers, pharmaceutical companies, and patients themselves. In terms of patient access, healthcare professionals have potential due to their ability to prescribe digital therapeutics. Other stakeholders’ potential stems from their capacity to influence healthcare professionals prescription decision. In terms of patient adherence, only healthcare professionals are of high relevance by onboarding patients and monitoring digital therapeutics use. Most healthcare stakeholders currently do not fully leverage their potential. Further educating healthcare professionals and simplifying processes for them, empowering patients to seek treatment with digital therapeutics, and designing digital therapeutics’ product features for better adherence can help improve patient access and adherence.

**Conclusions:**

Established healthcare stakeholders and digital therapeutics developers both need to take action to improve patient access and adherence to digital therapeutics. Several macro-level changes can support these efforts, including broader information dissemination, improved financial incentives, simplified prescription and activation processes, and a wider adoption of blended care and pay-for-performance payment models.

## Introduction

Mobile health (mHealth) applications are becoming increasingly accepted as an important component of future healthcare.^1^ Enabled by the proliferation of smart devices, connectivity, and computing power in recent years, they have the potential to improve access to care, optimize healthcare processes, improve clinical outcomes, and reduce the global burden of disease.^2,3^ Today, smartphone apps alone constitute 350,000 mHealth applications, with 250 added every day.^4^ These range from non-interventional, non-regulated consumer wearables for fitness monitoring, to prescribed and regulated digital therapeutics (DTx), which are the focus of this study. Although no universally agreed upon definition of DTx exists yet, DTx are frequently understood as applications that “deliver evidence-based therapeutic interventions that are driven by high-quality software programs to prevent, manage, or treat a medical disorder or disease.”^5^

While the efficacy of mHealth applications broadly is not (fully) established, DTx have been demonstrated to improve health outcomes for a variety of illnesses, including depression,^6^ diabetes,^7^ asthma, and COPD,^8^ amongst others. Nevertheless, adoption of DTx has been slow and cumbersome.^1^ This is often attributed to the lack of reimbursement, in addition to various social, technical, and procedural factors.^9–11^ To address these issues, in October 2020 Germany became the first country in the world to introduce DTx into standard care in the form of DiGA (“Digitale Gesundheitsanwendung,” German for “Digital Health Application”).^12^ Such applications must be based primarily on digital technologies, demonstrate (initial) positive care effects and have successfully applied for inclusion in the official DiGA directory in Germany.^13^

**Figure 1.**
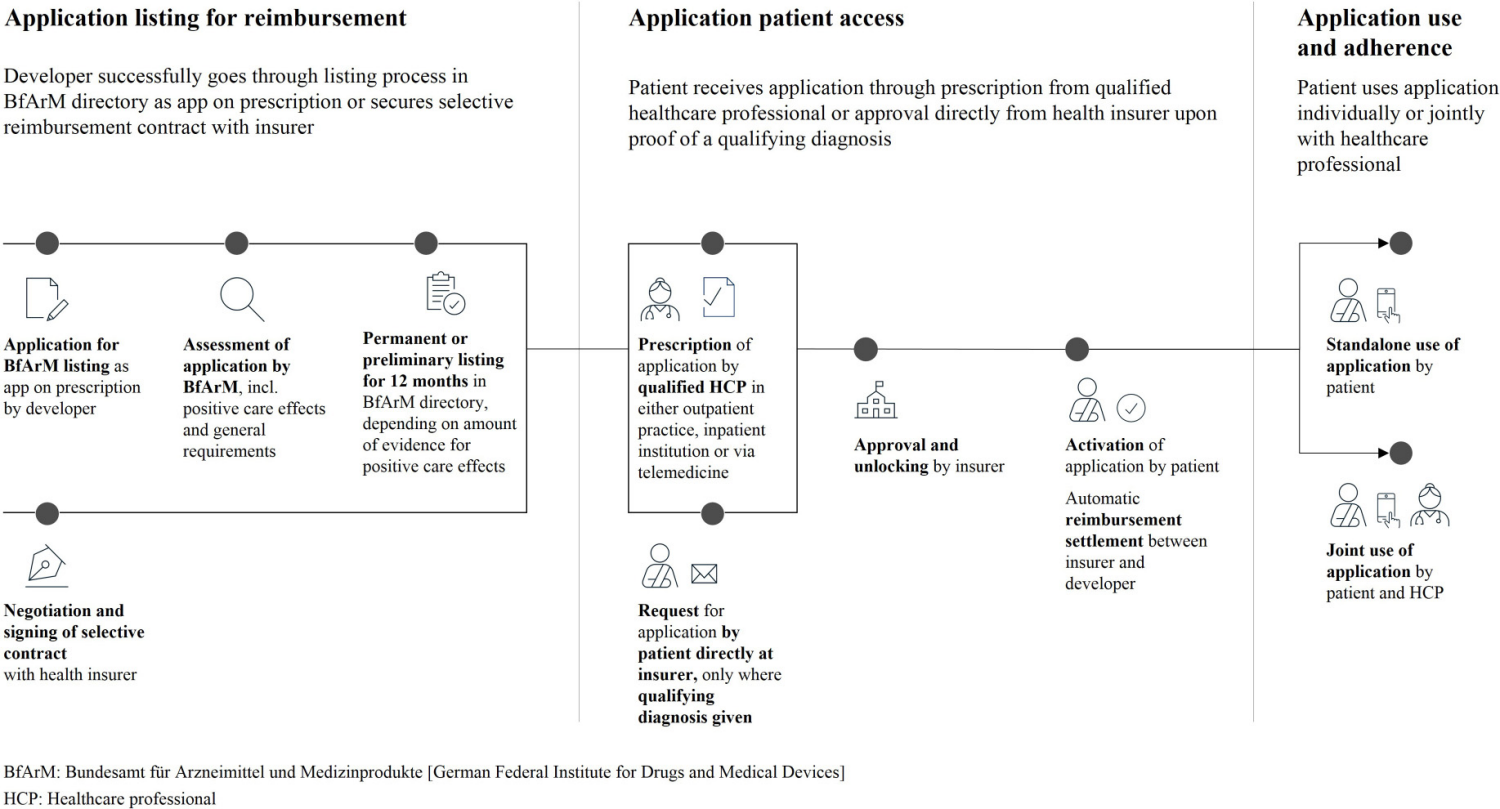
Schematic overview of process steps that DTx need to complete; from applying for reimbursement to securing adoption by physicians and insurers as well as ensuring subsequent patient use and adherence.^13,14^

As patients can receive application access either by prescription or after approval by their health insurer (Figure 1), healthcare professionals (HCPs) and health insurers play an important role in promoting the uptake of DTx in Germany.^15^ Yet, HCPs are reluctant to prescribe DTx,^16^ construing a barrier to patient access: Within the first 12 months after introduction, 14% of eligible HCPs had prescribed at least one DiGA, heavily concentrated in three medical specialties.^17^

Further barriers stem from suboptimal patient activation or adherence: Of the 50,100 DiGA prescribed (or approved directly by health insurers), only 78% were activated by patients.^17^ Additionally, past research on web-based health solutions suggests that program completion or adherence rates might be as low as 10%, with an average of 50%,^18^ similar to the adherence to traditional pharmaceuticals.^19^ This further prevents DTx from realizing their full potential for medical care.^18,20,21^

Given this situation, we set out to evaluate two questions: How can health systems provide patients with medically beneficial DTx, leveraging various healthcare stakeholders? And which approaches can be used to improve adherence to DTx treatment? Guided by these objectives, this article investigates how the medical potential of DTx can be realized at the healthcare system level in Germany. Despite some differences in health care systems, this also offers insights for healthcare actors in other countries that are seeking to integrate DTx into regular care, such as France, Belgium, Italy, and over six other European and North American countries.^10,22–24^

## Methods

### Design

We conducted an empirical qualitative study based on expert interviews with developers and distributors of DTx in Germany. The interviews comprised a qualitative and a quantitative part. In the qualitative part, open-ended questions were used to understand interviewees’ views on promoting access to DTx and patient adherence. The quantitative part used standardized closed questions to quantify respondents’ perceived relevance of each healthcare actor for these purposes. Responses were recorded on 10-point Likert-scales, with 1 and 10 corresponding to “no potential” and “very high potential,” respectively.

The original interview guide (see Supplemental material), containing semi-structured and structured components, was developed following a thorough literature review. It was piloted with nine experts from digital health and DTx companies (not included in the subsequent analysis), and subsequently reviewed and improved by the authors. The guideline included four broad areas:

- Details about the interviewee and application, such as target patient demographic, indications and patient pathways, and business model,
- Relevance of individual healthcare stakeholders for patient access and adherence,
- Strategies to promote patient access and adherence,
- Macro-factors to foster patient access and adherence

### Sampling strategy and recruitment of participants

To identify as many relevant DTx developers and distributors as possible, we screened various digital health databases (DiGA Directory, Bertelsmann Foundation Certified Medical Apps Database, and the German Industry Association for Digital Health members list^25–27^) for applications that qualify as DTx, and conducted press research for additional DTx developers and distributors. In order to ensure sufficient expertise in the local healthcare sector, only DTx from developer companies headquartered in Germany were considered. This yielded 47 distinct companies that had either already developed at least one DTx, were in the process of doing so or distributed DTx.

Inclusion criteria for experts from companies developing or distributing DTx were team- or board-leadership positions as well as professional expertise or practical experience with patient access to DTx. All participants received a cover letter with study details and a separate consent form for data use, which was signed by all interviewees.

### Data collection and analysis

Nineteen interviews across 19 distinct organizations were conducted between May and July in 2021 (Table 1). These interviews focused on DTx from a variety of conditions, including mental and behavioral disorders, musculoskeletal issues, neoplasms, diabetes, as well as various other areas. Interviews lasted between 30 and 90 min, with a median of 45 min. Seventeen interviews were conducted via video conference tools (i.e., Google Meet or Zoom) and two interviews were conducted via telephone. Interviews were audio recorded, transcribed verbatim, and analyzed inductively in line with grounded theory,^28–30^ using open, axial, and selective coding in MAXQDA software. Per the concept of theoretical sampling, data collection, analysis, and theory development were iteratively conducted until further interviews did not contribute to the research question further (i.e., until theoretical saturation was reached).

**Table 1.** Details of interviewees from expert interviews.

	Interviewee(s)	Organization	Application business model	Application purpose
#1	Chief Executive Officer	Startup	Insurer reimbursement, Corporate health management, B2B partnerships	Prevention & Screening, Treatment
#2	Chief Executive Officer, Head of Sales	Startup	Insurer reimbursement	Treatment
#3	Head of Business Development	Startup	Insurer reimbursement	Treatment, Aftercare
#4	Chief Executive Officer	Startup	Insurer reimbursement	Treatment
#5	Sales Manager	Pharma company	Insurer reimbursement	Treatment
#6	Chief Executive Officer	Startup	Insurer reimbursement, B2B partnerships	Treatment
#7	Managing Director	Startup	Insurer reimbursement	Treatment, Aftercare
#8	Project Manager	Pharma company	Insurer reimbursement	Treatment
#9	Project Manager	Pharma company	Insurer reimbursement	Treatment
#10	Chief Operating Officer	Startup	Insurer reimbursement	Treatment
#11	Co-Founder	Startup	Insurer reimbursement, Out-of-pocket	Treatment
#12	Project Manager	Pharma company	Insurer reimbursement	Treatment
#13	Business Development Manager	Startup	Insurer reimbursement, Corporate health management	Prevention & Screening, Treatment
#14	Chief Executive Officer	Startup	Insurer reimbursement	Treatment
#15	Managing Director	Startup	Insurer reimbursement, Corporate health management	Treatment
#16	Chief Executive Officer	Startup	Insurer reimbursement	Treatment
#17	Head of Sales	Startup	Insurer reimbursement	Treatment
#18	Co-Founder	Startup	Insurer reimbursement	Treatment
#19	Chief Executive Officer	Startup	Insurer reimbursement, B2B partnerships	Treatment

## Results

### Providers have the greatest potential to foster patient access and adherence according to DTx developers and distributors

**Figure 2.**
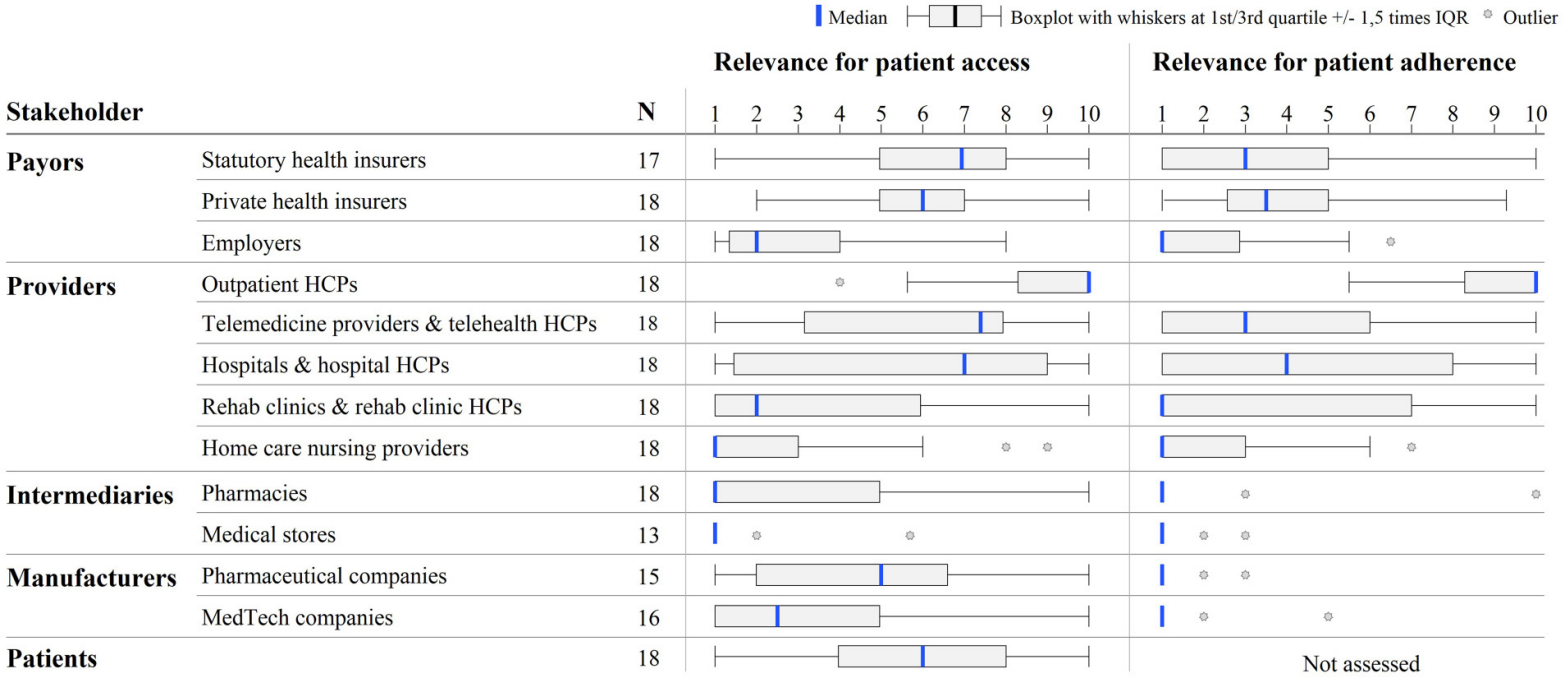
Relevance of stakeholders to foster patient access and adherence to DTx, clustered by stakeholder type, according to interviewed DTx developers and distributors.

A quantitative analysis of our interviews with DTx developers and distributors suggests that healthcare providers are seen as most important in promoting patient access to DTx, in particular HCPs who can prescribe DTx and are active in outpatient settings (median 10 out of 10), on telemedicine platforms (median 7.5) or in hospitals (median 7) (Figure 2). Some potential was also attributed to statutory and private health insurers (median 7 and 6, respectively), patients (median 6), and pharmaceutical companies (median 5). Several respondents also named medical associations and societies, physician networks, and practice management software developers as actors with large influence on patient access to DTx.

Apart from outpatient HCPs, all providers were rated as having a significantly lower potential for patient adherence when compared with patient access. In fact, only outpatient HCPs were considered to truly be able to promote adherence (median 10 out of 10), far ahead of HCPs on telemedicine platforms and in hospitals (median 3 and 4, respectively).

### While providers hold potential for patient access due to their ability to prescribe DTx, other stakeholders derive potential from their ability to influence providers

**Figure 3.**
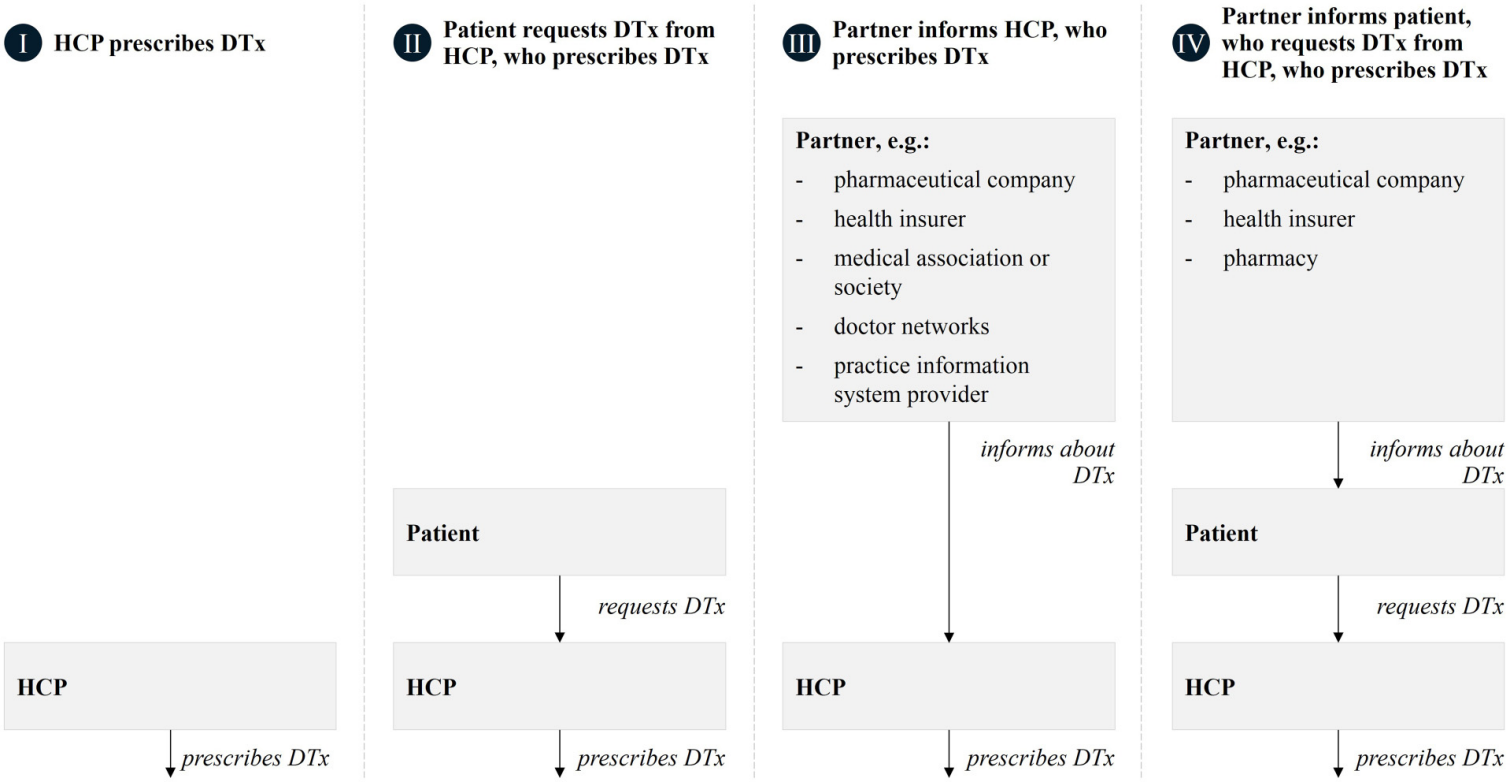
Four patient access pathways for digital therapeutics as identified from our interviews with DTx developers and distributors.

While respondents attributed the patient access potential of providers to their ability to prescribe DTx, other stakeholders’ potential—such as health insurers, pharmaceutical companies, medical associations and societies, doctor networks, practice information system providers and pharmacies—for promoting patient access was seen primarily in their direct and indirect (via patients) influence on providers (Figure 3).

### Direct effects on patient access

HCPs are regarded as relevant for DTx patient access due to their central gatekeeping role in the prescription process (see pathway I, Figure 3). This is further strengthened by trust from and influence on patients, especially in the outpatient sector characterized by longer-lasting patient–doctor relationships with frequent touchpoints.

*If the doctor says that's good, [the patient] will probably use it. If he says that's bad, then it's very likely that [the patient] won't use it. (Interviewee No. 19)*

At the same time, the role and importance of HCPs may vary by a DTx’ indication and resulting patient pathway. Interviewees stated that for some indications where inpatient treatment plays a central role in the patient journey, for example, rare or oncological diseases, inpatient HCPs are highly relevant to promote patient access. In most other cases, however, their potential is regarded as lower as they are restricted to prescribing DTx as part of release management.

*The problem with hospitals is that many [HCPs] cannot prescribe in hospitals. (Interviewee No. 17)*

The high potential of telemedicine providers for patient access was attributed to their availability, independent of time and day. This is especially relevant for DTx addressing indications associated with stigma, for example, erectile dysfunction, mental health issues, or obesity. Telemedicine's digital patient journey was also thought to reduce patient drop-out between prescription and DTx activation, solving a key issue in patient access for DTx.

*Our patients experience quite a lot of stigmatization, also by doctors […]. Which is why it is perhaps also quite pleasant for the patient to use a video consultation, because the hurdle for the patient to go to the doctor is still quite high. (Interviewee No. 16)*

However, providers overall are slow to adopt DTx and to support patient access, due to insufficient information, procedural hurdles, limited time and motivation, and inadequate financial incentives to prescribe DTx.

*There is an unbelievable lack of knowledge about digital health applications, and about the prescription process in general. (Interviewee No. 5)*

*[HCPs] have the possibility, but the technical processes with e-prescribing, e-prescription etc. are not yet up and running. (Interviewee No. 9)*

Patients’ potential to promote access to DTx was seen to be due to their ability to seek out their physician, psychotherapist, or insurer with a request for a DTx prescription or enrolment (see pathway II, Figure 3), particularly for illnesses with high patient awareness of illness and necessity of treatment, as well as high involvement and suffering pressure. Other interviewees, however, questioned the potential of such approaches, as patients would require HCP or insurer support for DTx access. Each patient would need to be informed individually, which may grow increasingly financially unviable and tedious over time with a growing number of DTx competing for patient attention.

*Addressing patients directly online works great. They get an overview with the 3-4 typical questions that we always hear [from doctors], take it to the doctor and it seems to work quite well, so that they actually get their doctor to prescribe it. The issue is that it's expensive as hell. You fight for every single patient. (Interviewee No. 16)*

### Other stakeholders’ indirect effects on patient access

The ability to educate HCPs about DTx earned various stakeholders potential for patient access (see pathway III, Figure 3). Several interviewees believe this to apply to insurers, given their large reach and influence, while others perceived limitations in insurers’ cost-conscious nature, and cautious approach to DTx. Pharmaceutical companies were believed to hold potential due to their existing relationships with providers that could help inform outpatient HCPs about DTx. However, other interviewees were sceptical, arguing that traditional pharmaceutical representatives may lack the necessary expertise, time, and incentives to explain digital products and support their integration into clinical practice.

*Pharma really opens up the outpatient sector. And for me, that's just it. They go door to door and know every doctor by name. (Interviewee No.* *19*)

*No doctor calls because of a pill, saying he can't get it into the patient's mouth. But if a doctor calls the [pharma] salesperson on the road [regarding DTx], and then such a banality comes up, then he stops working on digital. (Interviewee No. 15)*

Recommendations from HCP organizations like medical associations, professional societies, or doctor networks were also believed to hold potential given their large scale, trust by and influence on HCPs.

*If doctors see that [a DTx] is recognized as good by the professional associations, then you have a certain chance to create trust a little faster. (Interviewee No. 18)*

Integrated reminders of available and reimbursable DTx in patients’ files, via partnerships with practice management software providers, were also believed to improve patient access. Such collaborations could also provide HCPs with patient information materials, resulting in a higher share of patients activating their DTx.

The ability to inform eligible patients about DTx requests is a potential for various partners (see pathway IV, Figure 3). For example, insurers can utilize patient newsletters, position the DTx on the insurer website, or inform call center staff about the DTx. Similarly, pharmaceutical companies can bundle DTx with complementary traditional pharmaceutical products (“around-the-pill”-concepts^31^) and inform patients via packaging, with accompanying brochures or information campaigns.

*You approach the insurance company, so that they approach the patient themselves […] That's a possibility in a perfect world. But no insurance company will do so and offer a DTx, because it is simply a cost […], and as bitter as it is, they usually want to save on costs. (Interviewee No. 12)*

*I could also imagine that on certain products, such as herbal tranquilizers, they could put a barcode on the back that says: If you need more or want to get on top of your problems, check out [mental health DTx]. (Interviewee No. 13)*

Pharmacies hold potential in recommending selected DTx to patients or onboarding them for applications used together with medication that is frequently purchased from pharmacies. Other interviewees believed that the potential of pharmacies was limited by the rather transactional nature of pharmacy visits, lower trust in pharmacists than doctors and lack of mutually attractive partnership models, similar to approaches that involve patients’ employers and HCP groups who are not allowed to prescribe DTx themselves (e.g., physiotherapists).

*Pharmacies can be exciting if you have a pharmacist who says: OK, I see you have a pill here for high blood pressure. Have you ever thought about taking a DiGA on top of that? But the potential for patient access is strongly dependent on the indication. (Interviewee No. 16)*

### Developers and distributors of DTx believe that only providers hold large potential for patient adherence due to their ability to onboard patients and monitor DTx use

Providers are considered as key to patient adherence. HCPs active in outpatient, inpatient, and telemedicine settings may have similar effects on adherence by onboarding patients, for example, by answering patient questions or providing information materials, enthusiastically highlighting the patient's value of the DTx and generating motivation.

*Primary activation takes place through the therapist, who enthusiastically tells me about the advantage of DTx […]. If the doctor recommends something to me and I have a good feeling about it, then I do everything he says. Then compliance is already very high. (Interviewee No. 3)*

Their effect on adherence by accompanying the DTx use currently varies greatly, however: While inpatient and telemedicine physicians often lack recurring patient touchpoints after the DTx prescription, outpatient physicians have longer-term relationships with their patients, characterized by frequent touchpoints. Although currently not utilized in practice frequently, this theoretically increases their ability to monitor DTx use and request progress reports from patients, thereby increasing adherence.

*Doctors can be an institutionalized guilty conscience. Quote from a patient: In the beginning I didn't do this exercise for myself, but only for the coach, because I didn't want to […] say every time that I hadn't done anything, so I did it. And over time I realized that it makes sense and is really helpful. (Interviewee No. **7**)*

Other stakeholders were seen to hold less potential for adherence as interviewees doubted the existence of additional levers beyond those DTx developers could pull themselves. Only insurers, given their ability to financially incentivize both doctors, for example, to conduct follow-on checkups, and patients, for example, through premium refunds or by only fully reimbursing DTx costs in case of adherence—were perceived to have some potential.

### Several strategies can improve patient access and adherence and realize DTx benefits for patient care

**Figure 4.**
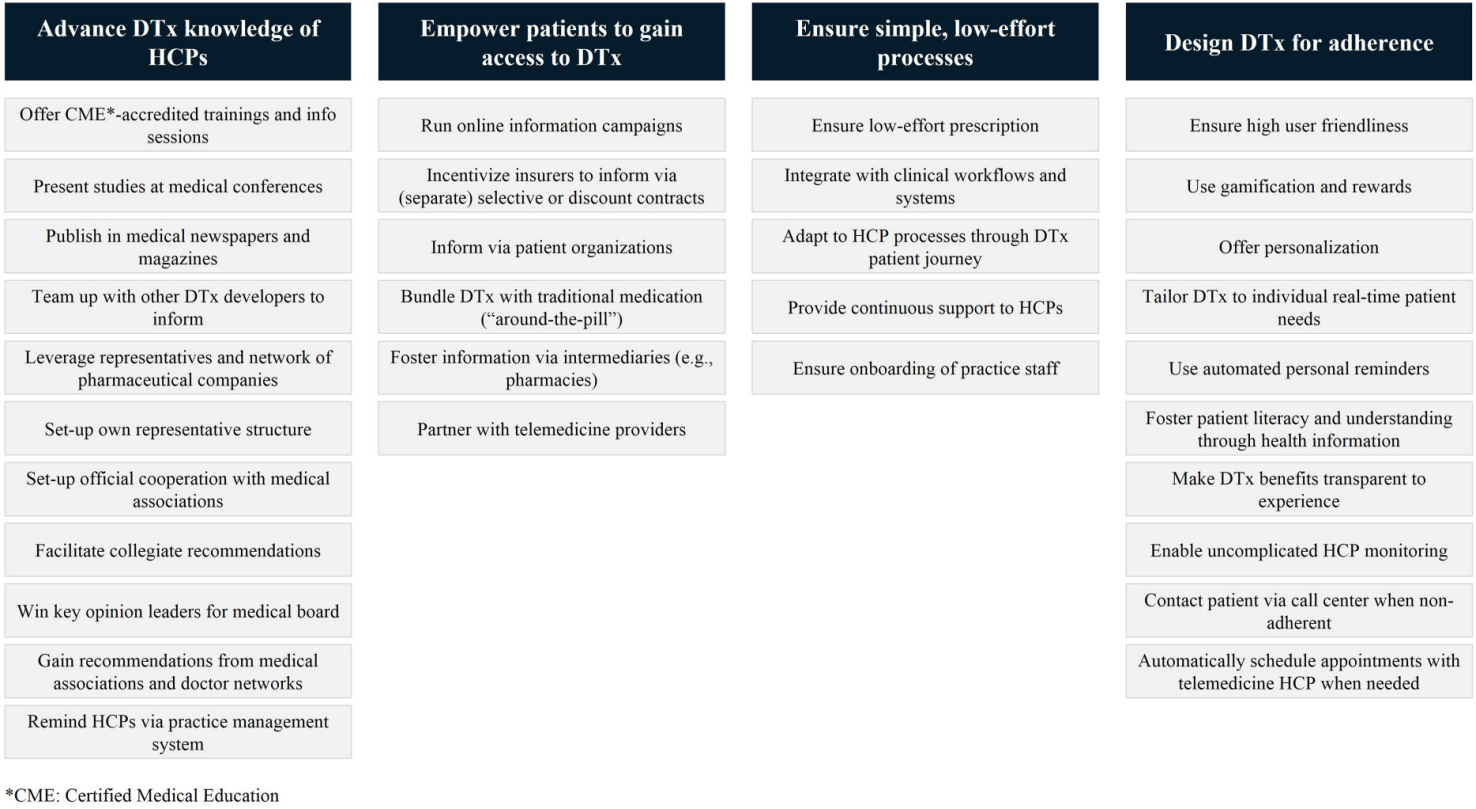
Four key themes derived from the coding of interview responses related to approaches for fostering patient access and adherence to DTx.

Despite the potential of these stakeholders to promote patient access and adherence to DTx, many were believed to currently realize only a small share of it. From the qualitative analysis of our interviews with developers and distributors of DTx, four key themes on how they attempt to foster access and adherence to realize the benefits of DTx for patient care emerged (Figure 4). These include:

- Advancing HCPs’ DTx knowledge and competencies, for example, through trainings, information materials, and press work; recommendations from key opinion leaders, especially those in medical organizations as well as distribution efforts, either by setting up own representative structures (especially where relevant patients are cared for by a limited number of specialists), by leveraging existing distribution networks of pharmaceutical companies or by sharing resources with other DTx developers.
- Empowering patients to gain access to DTx treatment, on the one hand by informing patients directly online, and on the other hand through partnerships with other stakeholders with existing patient touchpoints. The latter includes partnerships with health insurers (with information campaigns predominately implemented as part of selective or discount contracts), or via pharmaceutical companies or pharmacies, leveraging “around-the-pill”-concepts together with traditional medication.
- Ensuring simple, low-effort, and user-friendly processes for patients and prescribing HCPs that are adapted to existing practice operations and workflows. Additionally, onboarding of HCPs as well as practice staff and providing continuous support.
- Designing DTx for adherence, through both product features and ways of embedding DTx treatment in the broader care pathway. Developers particularly highlighted optimizing for user-friendliness, tailoring and personalization, gamification, reminders as well as ensuring patients understand the value of the DTx for their health as feature-related strategies to enhance patient adherence to recommended usage schedules. In addition, fostering HCP involvement in the DTx treatment process, for example by creating low-effort ways for prescribing HCPs to monitor DTx use, was seen to be critical for high adherence.

## Discussion

### Principal findings

Drawing on data from Germany as the first country worldwide to establish a national reimbursement pathway for DTx, this study extends the understanding of how DTx usage could gain more momentum through broader patient access and adherence.

Our results suggest that outpatient, inpatient, and telemedicine HCPs have the greatest potential to encourage patient access and widely disseminate digital health products, and to later ensure adherence. Although to a lower extent, health insurers, pharmaceutical companies as well as medical associations and societies, physician networks and practice management software providers, were also seen to hold potential for patient access due to their positive direct effect on HCPs or indirect effect via patients, adding to previous literature findings.^15^

However, our results also suggest that most of these stakeholders currently do not realize their full potential to foster patient access and adherence to DTx. While this is consistent with past research predominantly focused on clinicians’ adoption of general mHealth technologies,^11,32–35^ it also extends past literature findings to healthcare stakeholders more broadly. We identified key actions that DTx developers can take to address this situation and foster patient access and adherence to DTx. Building on suggestions by Bally and Cesuroglu as well as Gordon et al.,^15,36^ public health institutions, regulators, and other healthcare stakeholders can also facilitate those efforts through macro-level changes, to realize the full benefits of DTx for patient care.

### Recommendations to promote patient access

While our results suggest that HCPs are the main patient access gatekeepers, they reportedly also lack the knowledge and ability to integrate DTx into their practice, as demonstrated in earlier research.^16,37^ Therefore, educating and informing HCPs about DTx, reducing scepticism and fostering trust should be a priority, especially by influential healthcare stakeholders such as medical associations and societies, associations of statutory health insurance physicians and health insurers.^15^ Stronger medical evidence will play a vital role in winning the support of these stakeholders.^1^ Educating future physicians about DTx as part of the medical curriculum, and integrating treatment with DTx into medical guidelines, could support these efforts in the medium term.^38^

Past research suggests that HCPs should be seen as embedded within a broader adopter and organizational system.^39^ For instance, Greenhalgh's nonadoption, abandonment, scale-up, spread, and sustainability (NASSS) framework highlights, among others, how minimizing changes in staff roles, practices and professional identities and reducing the need for new team routines or care pathways can maximize likelihood of adoption. Similarly, previous research found HCP workflow fit, ease of use, and time savings to be critical for HCP adoption..^32,40^ Building on these findings, our results suggest that simplification and adaptation of prescription and accompaniment processes for DTx are needed to better fit existing routines and to foster adoption. Streamlined, fully digital e-prescription processes will be an integral part of this, lowering the barriers for ambivalent HCPs prescribing DTx but also for patients activating their prescription.^41^ Additionally, interoperability of DTx data with electronic patient records and existing clinical systems is key, enabling providers to easily monitor patients’ DTx use and associated benefits, which may provide positive feedback driving further adoption.^15,32,34^

Moreover, our results suggest that HCPs lack time and incentives to adopt DTx. This could be addressed by improving financial incentives for prescribing DTx, as well as monitoring use to increase patient access and adherence.^32^ These could be direct, for example, monetary compensation for prescription and progress checks, or indirect, for example, reduced visit frequencies of patients that do not result in additional compensation due to reimbursement rules.^42^

### Recommendations to foster patient adherence

Following DTx adoption, adherence has been argued to be critical to maximize DTx intervention efficacy.^43,44^ However, adherence to related web-based health systems has historically been low.^18^ Our results suggest that of all healthcare stakeholders surveyed, only HCPs have the potential to foster adherence.

Fostering the creation of hybrid or blended care models—that is, integrating online interventions with traditional care^45^—more broadly could help to leverage this potential better. Such approaches have been shown to increase patient adherence to DTx as well as patient access due to better acceptance by healthcare stakeholders.^46,47^ For DTx, this could involve face-to-face-consultations, calls from HCPs at developer-operated care centers upon detected patient non-adherence, or automatic consultations with telemedicine physicians as deemed necessary by DTx algorithms. Yet, legal barriers hinder realizing some of these approaches in Germany, for example, the requirement for DTx to be based on primarily digital technologies^13^ or limits to integration between DTx developers and providers, such as those active on telemedicine platforms.^48^

Given these challenges, DTx developers themselves play a critical role in fostering adherence through the design of their digital health solutions.^18^ Our results highlight that DTx developers already leverage several design principles that can improve adherence, such as personalization, gamification, and transparency.^18,20,44,49^ Past research, however, found that not all applications are optimized for adherence,^18^ resulting in weaker adherence and health impact, at unnecessary high costs to healthcare systems. Pay-for-performance or value-based care models could address this issue by providing stronger incentives for DTx developers to deliver high-quality DTx and continuously optimize with a focus on healthcare outcomes. Nevertheless, such approaches are not without weaknesses and challenges,^50,51^ for instance with measuring effect sizes and the true benefit of a DTx. Such models would also require more flexibility for DTx developers to continuously adapt their applications than is often the case, for instance in a manner similar to the FDA's Pre-Certification Pilot Program for DTx which shifts from episodic to continuous oversight.^52^

### Limitations and potential for future research

At the time of this study, only a limited number of DTx were available in Germany, posing a natural limit to this study's sample size. Further research may wish to expand on this study's findings to generate a more longitudinal overview of the role and importance of various stakeholders in promoting DTx access and adherence. Additionally, our interviews suggest that a DTx’ indication, patient pathway, and patient demographic may influence the relevance of individual stakeholders and strategies to foster access and adherence. Subsequent research may therefore wish to build on these findings to explore differences in individual DTx access and adherence strategies. Lastly, this study investigated healthcare stakeholders’ roles and potential for fostering patient access and adherence from the perspective of developers and distributors only. Given the relatively new concept of DTx, limited research has investigated strategies to foster adoption and adherence of DTx explicitly so far. Some studies have surveyed HCPs’ and patients’ attitudes towards DTx in general while no research has specifically investigated the perception of these stakeholders on access and adherence pathways and strategies for DTx.^1,16,53^ Further studies may therefore wish to expand upon this study and fill this gap in the current literature.

## Concluding remarks

This study highlights key stakeholders’ roles and potential for patient access and adherence to DTx and offers approaches for improvement. Recognition of these findings may be helpful to DTx developers, public health officials, and other healthcare stakeholders alike in creating an environment that enables DTx to thrive and ultimately realize their potential for patient care.

## Supplemental Material

sj-docx-1-dhj-10.1177_20552076221104672 - Supplemental material for There's an app for that, but nobody's using it: Insights on improving patient access and adherence to digital therapeutics in Germany Click here for additional data file. Supplemental material, sj-docx-1-dhj-10.1177_20552076221104672 for There's an app for that, but nobody's using it: Insights on improving patient access and adherence to digital therapeutics in Germany by Florian Dahlhausen, Maximillian Zinner, Linn Bieske, Jan P Ehlers, Philip Boehme and Leonard Fehring in Digital Health

## Abbreviations

BfArM: Bundesamt für Arzneimittel und Medizinprodukte [German Federal Institute for Drugs and Medical Devices]
CME: Certified Medical Education
DiGA: Digitale Gesundheitsanwendung [German state-certified digital health application]
DTx: digital therapeutic
HCP: healthcare professional.
