# Supplementary material for: There's an app for that, but nobody's using it: Insights on improving patient access and adherence to digital therapeutics in Germany
Source: Digit Health. 2022 Jul 3;8:20552076221104672. doi: 10.1177/20552076221104672 (PMC9260569; doi:10.1177/20552076221104672)
Supplement: sj-docx-1-dhj-10.1177_20552076221104672 - Supplemental material for There's an app for that, but nobody's using it: Insights on improving patient access and adherence to digital therapeutics in Germany [file sj-docx-1-dhj-10.1177_20552076221104672.docx]

**Interview guide for developers and distributors of digital therapeutics**

The interview guideline is presented to the interviewees in an open manner. Further questions arise from the answers. The questionnaire is structured as follows: A) Questions about interviewee and the company’s application(s) details, B) Strategies to get applications to patients and foster adherence, C) Relevance of individual stakeholders, D) Macro-level changes needed for better integration of digital therapeutics into care.

There are two types of questions: Open ended questions as well as semi-quantitative questions that require stating a value and an explanation why this value was chosen.

Part A: Details about the interviewee and digital therapeutic

- What is your role and what are your responsibilities regarding market- and patient access and marketing/sales?
- Which digital health application(s) does your company develop/distribute, and how are they certified (e.g., CE-mark, MDR/MDD, DiGA)?
- Are these applications intended for (primary) prevention, diagnosis/screening, therapy/coaching, or aftercare? *[if multiple: core purpose]*
- Who is your target patient or user?
- At what point in the patient journey does a patient or user first come into contact with your application(s)? *[if multiple: most frequent/important contact]*
- Do your application(s) realize their full potential for patients as standalone applications or do you require participation from other partners (e.g., HCPs) to realize it? If so, from whom and in what way?
- What is the business model for your application(s) in Germany/abroad? How has it changed over time?
- Have you listed your application(s) as DiGA or plan to do so? Why or why not?

Part B: Strategies to promote patient access and adherence

- How do you get your application(s) to patients in Germany and ensure patient access?
- How do you foster adherence to your application(s)?
- Which stakeholders have you approached or collaborated with so far to get your application(s) to patients or foster adherence?
- Which stakeholders would you consider approaching or collaborating with in the future to get your application(s) to patients or foster adherence?
- What do/could these efforts or collaborations look like?
- What is/could be the (mutual) benefit from these efforts/collaborations for both sides?
- What are the benefits and downsides of targeting/collaborating with these stakeholders?
- Which hurdles have you encountered in targeting/collaborating with these stakeholders, and how could these be overcome?
- When targeting/setting up a collaboration with these stakeholders, which factors are critical for success?

Part C: Relevance of healthcare stakeholders for patient access and adherence

For each stakeholder group included in Figure 2 in the main paper:

- On a scale from 1 to 10, what potential does *[stakeholder, seek Figure 2]* have for your patient acquisition, regardless of whether you (already) work with them or not (yet)? *[1 = no (potential) effect on gaining patients as users, 10 = very large (potential) effect on gaining patients as users]*
- Why have you assigned this value?
- On a scale from 1 to 10, what potential does *[stakeholder, seek Figure 2]* have for your patient adherence, regardless of whether you (already) work with them or not (yet)? *[1 = no (potential) effect on patient adherence, 10 = very large (potential) effect on patient adherence]*
- Why have you assigned this value?

Part D: Macro-factors changes needed

- How does ease of patient access to digital therapeutics in Germany compare to other countries?
- What are the biggest changes that are needed, by anyone, to better integrate digital health applications / digital therapeutics / DiGA into care in Germany?
